# Critical Illness Neuromyopathy Complicating Akinetic Crisis in Parkinsonism: Report of 3 Cases

**DOI:** 10.1097/MD.0000000000001118

**Published:** 2015-07-17

**Authors:** Margherita Capasso, Maria Vittoria De Angelis, Antonio Di Muzio, Francesca Anzellotti, Laura Bonanni, Astrid Thomas, Marco Onofrj

**Affiliations:** From the Neurology Clinic (MC, MVD, AD, FA, LB, MO), “SS Annunziata” Hospital; and Department of Neuroscience and Imaging (LB, AT, MO), University “G. d’Annunzio” of Chieti-Pescara, Chieti, Italy.

## Abstract

Akinetic crisis (AC) is a life-threatening complication of parkinsonism characterized by an acute severe akinetic-hypertonic state, consciousness disturbance, hyperthermia, and muscle enzymes elevation. Injectable dopaminomimetic drugs, high-dose methylprednisolone, and dantrolene are advocated as putative specific treatments. The course of the illness is frequently complicated by infections, pulmonary embolism, renal failure, disseminated intravascular coagulation, and cardiac arrhythmias.

Critical illness neuromyopathy (CINM) is an acquired neuromuscular disorder characterized by flaccid quadriparesis and muscle enzyme elevation, often occurring in intensive care units and primarily associated with inactivity, sepsis, multiorgan failure, neuromuscular blocking agents, and steroid treatment.

In 3 parkinsonian patients, during the course of AC we observed disappearance of rigidity but persistent hypoactivity. In all, neurological examination showed quadriparesis with loss of tendon reflexes and laboratory investigation disclosed a second peak of muscle enzymes elevation, following the first increment due to AC. Electrophysiological studies showed absent or reduced sensory nerve action potentials and compound muscular action potentials, myopathic changes, and fibrillation potentials at electromyography recordings, and reduced excitability or inexcitability of tibialis anterior at direct muscle stimulation, leading to a diagnosis of CINM in all 3 patients. In 1 patient, the diagnosis was also confirmed by muscle biopsy. Outcome was fatal in 2 of the 3 patients.

Although AC is associated with most of the known risk factors for CINM, the cooccurrence of the 2 disorders may be difficult to recognize and has never been reported. We found that CINM can occur as a severe complication of AC, and should be suspected when hypertonia-rigidity subsides despite persistent akinesia. Strict monitoring of muscle enzyme levels may help diagnosis. This finding addresses possible caveats in the use of putative treatments for AC.

## INTRODUCTION

Akinetic crisis (AC), also known as acute akinesia, is the most severe complication of Parkinson disease (PD) and related disorders, occurring with an annual incidence of 3 cases per 1000 parkinsonian patients.^1–3^ AC is a life-threatening condition characterized by an acute worsening of parkinsonian symptoms with a severe hypertonic-akinetic state, consciousness disturbance, dysphagia, dysautonomia, hyperthermia, muscle enzymes elevation, and transient unresponsiveness to dopaminergic treatment or to an increment of dopaminergic drug doses. Owing to clinical similarity with neuroleptic malignant syndrome, the disorder has also been termed as malignant syndrome, neuroleptic malignantlike syndrome, or parkinsonism-hyperpyrexia syndrome.^4–8^ AC is precipitated by infections, surgery, trauma, gastrointestinal disorders, inadvertent dopaminomimetic treatment's withdrawal, and admnistration of antidopaminergic drugs. Pneumonia and other infections, acute renal failure, venous thrombosis, pulmonary embolism, disseminated intravascular coagulation, and cardiac arrhythmias frequently develop during AC and may represent fatal complications.^1–8^ Management includes continuation of antiparkinsonian drugs, despite a lack of response, avoidance of antidopaminergic drugs, life supporting maneuvers, antithrombotic prophylaxis, and administration of putative specific treatments such as bromocriptine, amantadine sulphate, apomorphine, dantrolene, and methylprednisolone.^2,3,9–14^

An acute neuromuscular dysfunction with flaccid quadriparesis and difficulty to weaning from mechanical ventilator, with or without sensory disturbances, often occurs in patients admitted to intensive care units (ICUs). Electrophysiological and pathological studies demonstrated primary involvement of muscles, nerves, or both leading to define 3 nosological entities: critical illness myopathy, critical illness neuropathy, and critical illness neuromyopathy (CINM).^15,16^ Main risk factors include inactivity, sepsis or systemic inflammatory response syndrome (SIRS), multiorgan failure, neuromuscular blocking agents, and corticosteroids.^15–17^

In this report, we recognize CINM as a further possible complication of AC. We also examine possible pathophysiological mechanisms underlying the cooccurrence of the 2 disorders and discuss implications for treatment.

## CASE REPORTS

### Case 1

**FIGURE 1:**
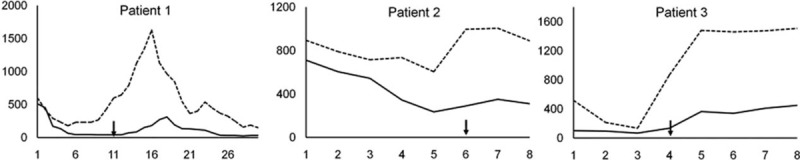
Muscle enzymes trends in 3 patients with critical illness neuromyopathy during akinetic crisis. In abscissae: days from admission. In ordinates: levels of creatine kinase (U/L) and myoglobin (mcg/L, dotted lines). Arrows indicate the appearance of flaccid quadriparesis.

**FIGURE 2:**
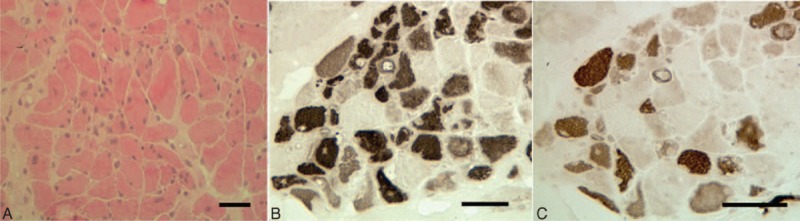
Case 1. Biceps brachii biopsy: 7 μm cryostat serial sections stained with hematoxylin–eosin (A), ATPase pH 10.4 (B), and pH 4.6 (C). Bar = 50 μm. Note the reduction in myosin ATPase reactivity in many nonnecrotic myofibers, indicating myosin loss typical of critical illness myopathy.

A 71-year-old man, affected by PD for 15 years and arterial hypertension, was treated with deep brain stimulation of the subthalamic nuclei, levodopa (750 mg/d), amantadine (100 mg/d), clozapine (50 mg/d), and enalapril (20 mg/d). He was admitted to another hospital for intestinal subocclusion and treated with evacuative enemas and intramuscular clebopride, an antidopaminergic gastrointestinal prokinetic drug. In few days, his neurological status worsened, hyperpyrexia developed (38.5°C), and he was transferred to our Neurology Clinic. On admission, he presented with drowsiness, severe akinesia and rigidity, marked dysphonia, and dysphagia requiring nasogastric tube placement for feeding and drug administration. Muscle strength was preserved. Unified Parkinson's Disease Rating Scale (UPDRS) motor score was 61 and was increased by 31 points compared to the last outpatient evaluation performed 4 months before. Overall, akinesia items (finger tapping, hand movements, pronation–supination hands, toe tapping, leg ability, and body bradikinesia) were increased by 13 points. Creatine kinase (CK) and myoglobin were 510 U/L (normal value 30–170) and 596 mcg/L (normal value 16.3–96.5), respectively. Chest x-rays showed right pneumonia. Laboratory investigations showed hypoalbuminemia (1956 mg/dL; normal value 3500–5000) and mild normochromic normocytic anemia. We withdrew clozapine and started continuous subcutaneous infusion of apomorphine (100 mg/d) with ondansetron (8 mg/d), intravenous ceftriaxone and ciprofloxacin, subcutaneous enoxaparin (4000 IU/d), and oxygen delivery via nasal cannula (2 L/min). Methylprednisolone (500 mg/d) was also administered for 5 days. By day 5, fever subsided, CK normalized, myoglobin fell to 177 mcg/L, and full recovery of consciousness occurred with minor improvement of rigidity and akinesia. On day 11, rigidity subsided but the patient continued to be markedly hypoactive. Neurological examination showed flaccid severe quadriparesis with loss of tendon reflexes in the absence of overt sensory loss. Strength was grades 2 to 3 of the Medical Research Council (MRC) Scale on triceps and biceps muscles, and grades 1 to 2 on the remainder muscles of upper and lower limbs. Myoglobin raised up to 1627 mcg/L in 5 days and then progressively decreased (Figure 1). On day 16, electromyography showed few fibrillation potentials in proximal and distal muscles, and early recruitment of short duration, low amplitude motor unit potentials in biceps brachii. Neurography showed reduced sensory nerve action potentials and reduced compound muscular action potentials (CMAPs) in upper and lower limbs. Direct muscle stimulation of the right tibialis anterior (TA) performed according to Trojaborg et al^18^ revealed a low amplitude potential (0.4 mV; ratio between CMAP obtained after peroneal nerve and TA stimulation =0.5). Apomorphine was withdrawn and rehabilitation was started. Left biceps brachii biopsy, performed on day 32, confirmed the diagnosis of critical illness myopathy with myosin loss, showing pronounced fiber size variability, atrophy of both type 1 and type 2 fibers, scattered and grouped angulated fibers, some necrotic fibers, and patchy or complete reduction in myosin ATPase reactivity in many nonnecrotic muscle fibers (Figure 2). During the following months, dysphagia improved and nasogastric tube was removed, muscle strength gradually normalized, and the patient became able to walk unaided. At the outpatient evaluation performed 10 months after the hospitalization, UPDRS motor score was 33.

### Case 2

An 84-year-old lady, affected by type 2 diabetes mellitus, arterial hypertension, and dementia with Lewy bodies for 3 years, was admitted for fever (up to 39°C) and drowsiness 10 days after right leg fracture. At home she was treated with metformin (1500 mg/d), enalapril (5 mg/d), levodopa (300 mg/d), quetiapine (50 mg/d), citalopram (20 mg/d), amoxicillin (2 g/d), and subcutaneous parnaparin (4250 IU/d). Until 4 days before admission the patient was not allowed to walk because of the recent leg fracture, but she was reported by her relatives to speak clearly and able to sit and feed herself unaided. Before leg trauma she was reported to walk unsupported. On admission, she showed generalized severe rigidity, akinesia, dysphonia, and severe dysphagia. Muscle strength was preserved. UPDRS motor score was 55 (with 24 points coming from akinesia items). Blood pressure was 95/60 mm Hg. Laboratory investigation revealed leukocytosis (white blood cell 18,200) and high erythrocyte sedimentation rate (62 mm/h). CK and myoglobin levels were 710 U/L and 894 mcg/L, respectively. Brain computed tomography (CT) scan was unremarkable. Electroencephalography showed diffuse slow activity. Cerebrospinal fluid (CSF) examination was normal except for mild protein increase (68 mg/dL). Nasogastric tube was placed, and enteral nutrition, intravenous hydration, intravenous ceftriaxone, and melevodopa/carbidopa (100/25 mg 3 times daily) were started. Quetiapine and citalopram were withdrawn. Although the clinical picture indicated a sistemic inflammatory response syndrome, further investigations failed to disclose an associated infection, namely blood, CSF, bronchoaspirate and urine cultures, chest x-rays, and echocardiography were normal. Bone x-rays showed no signs of osteomyelitis. By the third day, sustained hypotension required treatment with intravenous methylprednisolone and continuous intravenous infusion of dopamine. On day 6, rigidity subsided despite persistent inactivity and quadriplegia with loss of tendon reflexes was recognized. An increase in muscle enzyme levels was noticed (Figure 1). Electrophysiological examination showed fibrillation potentials in leg muscles, inexcitability of peroneal nerves, and markedly reduced CMAP and sensory action potential of the right median nerve. At direct muscle stimulation, recording with a concentric needle electrode, right TA was inexcitable. The patient died 2 days later from sudden cardiocirculatory arrest.

### Case 3

An 85-year-old lady, affected by PD for 5 years, was admitted for consciousness alteration and fever (up to 38°C) 6 days after an upper respiratory tract infection, followed by drowsiness and consequent withdrawal of dopaminergic treatment (levodopa 300 mg/d). At the last outpatient visit, 6 months before, UPDRS motor score was 37. However, 1 month before admission she had experienced left femoral fracture and had not restarted to stand and walk, but she was reported to speak clearly and to sit and feed herself unaided. On admission, she showed generalized rigidity, akinesia, and severe dysphagia. Muscle strength was preserved. UPDRS motor score was 63, with an increment in akinesia items by 11 points. Blood pressure was 100/50 mm Hg. CK and myoglobin were 102 U/L and 517 mcg/L, respectively (Figure 1). Chest x-rays showed right pneumonia with pleural effusion. Brain CT scan was unremarkable. Blood, bronchoaspirate, and urine cultures were normal. We started continuous subcutaneous infusion of apomorphine (100 mg/d) with ondansetron (8 mg/d). Nasogastric tube was placed, and enteral nutrition and melevodopa/carbidopa (100/25 mg 6 times daily) were started. Intravenous hydration, ceftriaxone, methylprednisolone (500 mg/d), and subcutaneous fondaparinux (2.5 mg/d) were also administered. She developed sustained hypotension requiring treatment with continuous intravenous dopamine infusion. By day 3, fever subsided, myoglobin fell to 134 mcg/L, and consciousness recovered. On day 4, the patient was found markedly hypoactive despite disappearance of rigidity. Neurological examination showed severe quadriparesis (MRC grades 1–2 in upper limb muscles, grades 0–1 in lower limb muscles) and loss of tendon reflexes. Myoglobin raised up to 1483 mcg/L in 2 days (Figure 1). Electrophysiological examination showed early recruitment of low amplitude short duration motor unit potentials in biceps brachii, absence of muscle activity in leg muscles, inexcitability of peroneal nerves, and inexcitability of the right TA at the direct muscle stimulation. One day 7, the patient became anuric and her consciousness worsened. She died 1 day later from cardiocirculatory arrest.

All interventions given were part of normal health care, and thus ethical approval was not necessary. We obtained informed consent from patient 1 and relatives of patient 2 and patient 3 for using their clinical data for publication and teaching purposes.

## DISCUSSION

We would first remark the key elements for the diagnosis of AC in the three cases we report, with particular regard to the distinction from neuroleptic malignant syndrome in patient 1 and to the precipitating role of levodopa withdrawal in patient 2 and patient 3. Neuroleptic malignant syndrome and AC share a common clinical picture and probably represent 2 facets of the same disorder. By definition, neuroleptic malignant syndrome is related to the administration of neuroleptics or D2 receptor antagonist, such us metoclopramide, used for nonpsychiatric diseases. Conversely, AC is an acute worsening of a preexisting parkinsonism associated with transient unresponsiveness to dopaminomimetic treatment. Operatively, we defined AC as a sudden worsening of UPDRS motor score by at least 20 points accompanied by at least 3 days of unresponsiveness to a previously effective dopaminomimetic treatment or to dopaminergic rescue drug administration.^3^ In some patients, AC may be precipitated by drug manipulations, such us levodopa withdrawal/reduction or exposure to antidopaminergic drugs. In case 2 and case 3, the worsening of parkinsonian symptoms was preceded by levodopa withdrawal, but it was not promptly reversed by reintroduction of levodopa or apomorphine administration, and thus it was not considerable as a mere consequence of treatment withdrawal but due to AC. When antidopaminergic drug administration precedes the onset of clinical deterioration in parkinsonism, as in patient 1, it may be questionable to classify the disorder as AC or neuroleptic malignant syndrome. We found more appropriate to consider this case as AC because of the preexisting parkinsonism, the cooccurrence of at least another putative precipitating factor, such as ileus, and because of the characteristic transient refractoriness to antiparkinsonian drugs. Japanese authors, who prefer to term AC as “malignant syndrome,” as well consider this condition as distinct from “neuroleptic malignant syndrome” because, in the former, parkinsonism is the underlying disease, even though neuroleptic administration may constitute a precipitating event in some cases.^19–21^

Immobility, sepsis, multiorgan failure, and administration of high-dose corticosteroids commonly occur during AC and represent multiple risk factors for critical illness neuromuscular dysfunction.^15–17,22–23^ Therefore, our finding that parkinsonian patients may develop CINM during AC should not be surprising. Nevertheless, this complication was not previously reported, possibly because of the high index of suspicion required to diagnose CINM outside the traditional setting of ICUs. Indeed, previous prospective studies have shown that 25% to 40% of patients admitted to ICUs, mostly for sepsis, SIRS, or multiorgan failure, with or without acute lung injury, developed clinical evidence of a neuromuscular disorder.^16^ Conversely, CINM has been only anecdotally reported in medical and surgical departments, and there are no precise estimates of its occurrence outside the usual setting of ICUs.^24^ However, neuromuscular dysfunction frequently appears during the early stages of critical illness and it is likely that CINM develops unrecognized in a relevant percentage of severely ill patients not requiring ICU admission or before their admission in the ICU. In the 3 patients we report, we could suspect CINM because of the persistence of akinesia despite the disappearance of rigidity and because of a diphasic pattern of muscle enzymes increments (Figure 2). However, when rigidity subsides in patients recovering from AC, especially when consciousness disturbance persists, it may be difficult to recognize if motor impairment is due to residual akinesia or to an overlapping neuromuscular disorder. Moreover, muscle enzymes elevation, occurring both in AC and CINM, may not help diagnosis unless strictly monitored. Our findings suggest that, during AC, patients should be strictly observed for the appearance of clinical signs of neuromyopathy, which should suggest prompt electrophysiological examination, and that muscle enzymes levels should be daily monitored.

Pathophysiological mechanisms of CINM are incompletely understood. Weakness may origin from different structural or functional changes including atrophy or necrosis of muscle fibers, loss of myosin, muscle inexcitability, and axonal degeneration or inexcitability.^17,22–23^ At a molecular level, these changes have been related to microvascular disfunction, oxidative stress, production of inflammatory cytokines, nitric oxide synthase abnormalities, activation of apoptotic pathways, decreased myosin transcription rate, and sodium channel inactivation.^22,22–23,25–31^ Moreover, mitochondrial dysfunction contributes to impair muscle function because of energy depletion.^32–34^ Indeed, previous work has shown that concentration of ATP and creatine phosphate and activities of citrate synthase and of respiratory chain complexes I and IV are decreased in muscle samples obtained from patients with sepsis and multiple organ failure.^32–33^ A decrement in citrate synthase concentrations has also been found in muscle homogenates obtained from critically ill patients during the early course of CINM.^34^ More recently, in an animal model of sepsis, a reduction in the efficiency of oxidative phosphorylation in the mitochondria and reduced levels of cytochrome c oxidase have been found in brain homogenates.^35–37^ Thus, mitochondrial abnormalities play a role in both muscle and central nervous system derangement during sepsis. On the contrary, mitochondrial dysfunction is also emerging as a key mechanism in the pathophysiology of AC. It has been known for a long time that patients with PDs show a decreased activity of respiratory chain complex I in the substantia nigra.^38^ Recently, we have described recurrent or fatal AC in parkinsonian patients carrying mutations in POLG1 and PINK1, 2 genes associated with monogenic parkinsonism and encoding, respectively, for the catalytic subunit of mitochondrial DNA polymerase and for a mitochondrial serine/threonine-protein kinase involved in cell protection from stress-induced mitochondrial dysfunction.^39^ Moreover, we evaluated 5 patients with AC by FP/CIT single-photon emission computerized tomography and found a dramatic loss of striatum dopamine transporter-binding. We demonstrated that this finding was not related to an effect of dopaminomitetic drugs and indicated as an alternative explanation a failure in energetic metabolism.^40^ Of note, patients with PDs also show a decreased activity of respiratory chain complexes I and IV in skeletal muscle.^41–42^ More recently, immunohistochemical studies in a patient who died from neuroleptic malignant syndrome, a disorder akin to AC or a facet of the same disorder in which akinesia-hypertonia is induced by neuroleptics, have shown loss of mitochondria in skeletal muscle.^43^ Overall, these data suggest that mitochondrial dysfunction may play a pivotal role in the cooccurrence of AC and CINM.

Although the general management of both conditions may appear quite similar, we think it is clinically relevant to recognize the occurrence of CINM during the course of AC for several reasons. First, the cooccurrence of CINM changes the prognosis, as recovery from CINM requires several months and prolonged rehabilitation. Rehabilitation strategies should be targeted to muscle strength recovery and not solely to parkinsonian symptoms and disability induced by prolonged immobility. Moreover, specific therapies for CINM have been proposed and are still object of ongoing clinical investigations, such us electrical muscle stimulation, nutritional interventions, antioxidant therapy, testosterone derivates, growth hormones, and immunoglobulins.^44^ Further specific therapeutical strategies could be available in the future with the increasing knowledge of pathophysiological mechanisms of CINM. As a final argument, we should focus on putative treatments for AC. High-dose corticosteroids are frequently used in treating AC as the only existing double blind study on AC therapy suggests that their administration improves outcome.^14^ Dantrolene administration, because of the similarity of symptoms in AC and in Malignant hyperthermia, is also currently advocated by some authors as a possible treatment for the disorder.^11–13^ Yet, both treatments are considered as precipitating factors for CINM. Therefore, our findings call for caution on the traditional pharmacological approach to AC. Conversely, a therapeutical strategy targeting mitochondrial protection should be considered.
